# The transcription factor LEF1 promotes tumorigenicity and activates the TGF-β signaling pathway in esophageal squamous cell carcinoma

**DOI:** 10.1186/s13046-019-1296-7

**Published:** 2019-07-11

**Authors:** Yue Zhao, Ji Zhu, Bowen Shi, Xinyu Wang, Qijue Lu, Chunguang Li, Hezhong Chen

**Affiliations:** Department of Thoracic Surgery, Changhai Hospital, Second Military Medical University, Shanghai, 200433 China

**Keywords:** OV6, Lymphoid enhancer-binding factor 1 (LEF1), Esophageal squamous cell carcinoma (ESCC), Prognosis, CSC-like phenotype

## Abstract

**Background:**

Esophageal squamous cell carcinoma (ESCC) is the most difficult subtype of esophageal cancer to treat due to the paucity of effective targeted therapy. ESCC is believed to arise from cancer stem cells (CSCs) that contribute to metastasis and chemoresistance. Despite advances in diagnosis and treatment, the prognosis of ESCC patients remains poor.

**Methods:**

In this study, we applied western blot, quantitative real-time polymerase chain reaction (qRT-PCR), immunohistochemistry, RNA-Seq analysis, luciferase reporter assay, Chip-qPCR, bioinformatics analysis, and a series of functional assays to show the potential role of LEF1 in regulating esophageal CSCs.

**Results:**

We found that the overexpression of LEF1 was associated with aberrant clinicopathological characteristics and the poor prognosis of ESCC patients. In addition, the elevated expression of LEF1 and OV6 was significantly associated with aberrant clinicopathological features, and poor patient prognosis. Moreover, the overexpression of LEF1 was observed in esophageal CSCs purified by the magnetic sorting of adherent and spheroidal ESCC cells. The increased level of LEF1 in CSCs facilitated the expression of CSC markers, stem cell-like properties, resistance to chemotherapy, and tumorigenicity and increased the percentage of CSCs in ESCC samples. Conversely, the knockdown of LEF1 significantly diminished the self-renewal properties of ESCC. We showed that LEF1 played an important mechanical role in activating the TGF-β signaling pathway by directly binding to the ID1 gene promoter. A positive association between LEF1 and ID1 expression was also observed in clinical ESCC samples.

**Conclusion:**

Our results indicate that the overexpression of LEF1 promotes a CSC-like phenotype in and the tumorigenicity of ESCC by activating the TGF-β signaling pathway. The inhibition of LEF1 might therefore be a novel therapeutic target to inactivate CSCs and inhibit tumor progression.

**Electronic supplementary material:**

The online version of this article (10.1186/s13046-019-1296-7) contains supplementary material, which is available to authorized users.

## Background

Esophageal carcinoma is the eighth most common cause of cancer-related deaths worldwide [1]. Esophageal squamous cell carcinoma (ESCC) is the most important histopathological form of esophageal carcinoma [2]. Despite advances in diagnosis and therapy, the prognosis of patients with ESCC remains poor even after surgery [3]. ESCC is also a highly aggressive malignancy due to the distant metastasis and invasion of neighboring organs [4].

Cancer stem cells (CSCs) are known to exist in different types of cancers and contribute to aggressive tumor behavior [5]. CSCs are responsible for tumorigenicity and resistance to therapies such as chemotherapy and radiotherapy after surgery. Recently, it was reported that CSCs from ESCC can be identified by several cell markers, including CD133 and CD90 [6, 7]. OV6, an epithelial origin marker, was first found to be abundant in hepatic progenitor cells and bile epithelial cells [8] and was then identified as a CSC surface marker and correlated with tumor progression in hepatocellular carcinoma [9, 10]. In our previous study, we demonstrated that OV6 expression was also closely associated with the clinical outcome and prognosis of ESCC patients and contributed to tumorigenesis and chemotherapy resistance [11]. However, the mechanisms involved in the expansion and function of CSCs are not well understood.

Lymphoid enhancer-binding factor 1 (LEF1), a member of the T-cell factor (TCF)/LEF1 family of high mobility transcription factors, is predominantly involved in the Wnt/β-catenin signaling pathway [12]. Several studies indicated that LEF1 was overexpressed in lung adenocarcinomas and oral squamous cell carcinoma, and its aberrant expression was closely associated with tumor progression and poor prognosis [13, 14]. It has also been reported that the Wnt signaling pathway regulates cancer stem cell-like properties and contributes to the tumorigenic properties of many cancers, including breast cancer, esophageal cancer, etc. As a downstream mediator of the Wnt signaling pathway, LEF1 is also essential for stem cell maintenance and organ development in addition to its role in epithelial-mesenchymal transition (EMT) by activating the transcription of markers [15, 16]. Moreover, epithelial cancer cells can obtain stem-like properties through EMT, a process that also plays a crucial role in cancer progression [17]. Our previous study has also shown that LEF1 is upregulated in ESCC and could positively regulate the invasion, migration, and EMT of ESCC through the cooperation of the OCT4 transcription factor, which indicates the potential role of LEF1 in mediating the self-renewal properties of CSCs [18]. However, the role of LEF1 in the transcriptional regulation of CSC regulators during ESCC progression remains unclear.

The transforming growth factor beta (TGF-β) signaling pathway is a critical regulator involved in cell growth, differentiation, and development [19]. The aberrant activation of the TGF-β signaling pathway is responsible for the self-renewal properties and drug resistance of various cancers, including hepatocellular carcinoma, esophageal squamous cell carcinoma, and colorectal cancer [20–22]. During the initiation and progression of tumors, TGF-β could facilitate EMT, a process that is crucial for the acquisition of CSC-like properties [23]. Thus, the specific inhibition of TGF-β signaling has been developed for anti-cancer therapy. A recent study reported that the application of a TGF-β inhibitor prevents the development of CSCs, which promotes the chemotherapeutic effect against triple-negative breast cancer [24]. Taken together, these data suggest that hyperactivation of the TGF-β signaling pathway leads to tumorigenicity and stem-like properties of CSCs in various tumors. Inhibitor of DNA binding 1 (ID1), a member of the ID protein superfamily, belongs to the helix-loop-helix transcription factor family. ID1 is widely expressed in many tissues and functions in a number of biological processes, including cell proliferation and apoptosis, among others [25]. Many studies indicate that ID1 is an oncogene and is critical in promoting cancer progression. High levels of ID1 are found in human cancers of the breast, lung, and esophagus and are associated with poor patient prognosis [26]. In the process of cell development, ID1 plays an important role in the maintenance of embryonic stem cell self-renewal and hematopoietic stem cells [27, 28]. ID1 has also been identified as a key regulator of the CSC phenotype in colon cancer [29]. Furthermore, the important role of ID1 in CSC phenotypes and its participation in the TGF-β-SMAD2/3-ID1 axis make it a candidate for the study of cancer stem cell properties in ESCC.

In the present study, we report that LEF1 overexpression promotes TGF-β signaling pathway activation by directly binding to ID1 to enhance tumorigenesis and the CSC-like phenotype of ESCC in vitro and in vivo. These results provide novel insight into LEF1-mediated tumorigenesis and the mechanisms by which the TGF-β signaling pathway is activated in ESCC.

## Methods

### Patients and tissue specimens

The tissue microarrays chips (TMA) of the ESCC cohort (31 patients, 98 patients, 75 patients and 70 patients) were purchased from Shanghai Weiao Biotech Company and Wuhan Servicebio Biotech Company, which were obtained from 4 different medical centers. All specimens were well documented with complete follow-ups for periods from 4 to 5 years. Ninety-five patient specimens were collected from patients who were diagnosed with primary ESCC and who received radical esophageal surgery without preoperative chemoradiotherapy from 2012 to 2013 at Changhai Hospital (Shanghai, China). All samples were fixed in 4% formaldehyde and embedded in paraffin wax. The patient samples were obtained with informed consent according to an established protocol approved by the Ethics Committee of Changhai Hospital. 95 patients in our center were observed until May 2017, with a median observance time of 27 months.

### Cell culture

The human Eca109, TE1 cells were purchased from the Shanghai Cell Bank (Shanghai, China). After measured by mycoplasma detection, DNA-Fingerprinting, isozyme detection and cell vitality detection, these cell lines were immediately expanded and frozen such that they could be restarted every 3 to 4 months. All cell lines cultured in Dulbecco’s modified Eagle’s medium (Gibco, CA, USA) supplemented with 10% heat-inactivated foetal bovine serum (Gibco-BRL) and antibiotics (100 U/ml penicillin and 100 U/ml streptomycin, HyClone Laboratories, Inc., USA) at 37 °C in a humidified atmosphere of 5% CO2. Spheroids were cultured in F12/DMEM (Gibco, CA, USA) supplemented with EGF (Sigma, St Louis, USA), FGF (Gibco, CA, USA) and ITS (Sigma, St Louis, USA).

### Vector construction and lentivirus infection

We constructed lentiviral vectors encoding the human LEF1 gene or green fluorescent protein (GFP) in the pLenti-EF1a-EGFP-P2A-Puro-CMV-MCS-3Flag vector (HeYuan Bio-technology Co., Shanghai, China) and designated them as LV-LEF1 or LV-GFP. The lentiviral vectors were transfected into the HCC cells with a multiplicity of infection (MOI) 5 in the presence of polybrene (5 μg/ml) for 6 h. Stable Eca109 and TE-1 cells knockdown of LEF1 were generated using lentiviral constructs expressing shLEF1(shLEF1^1#^CCCATCCCGAGAACATCAA; shLEF1^2#^CCTCATCCAGCTATTGTAA; shLEF1^3#^GCTACATATGCAGCTTTAT) and negative control (HeYuan Bio-technology Co., Shanghai, China), and incubated with 2 μg/ml puromycin (Sigma, St Louis, USA).

### Magnetic cell sorting and flow cytometry

Cells were labelled with primary OV6 antibody (mouse IgG1; R&D Systems, Minneapolis), magnetically tethered to rat anti-mouse IgG1 microbeads, and sorted with a Mini-MACS™ Cell Sorter Kit (Miltenyi Biotec, CA). All of the procedures were following the manufacturer’s instructions. The sorted cells were evaluated by flow cytometry analysis. The flow cytometry was performed with MoFlo Sorter (Beckman, CA) or ImageStream^x^ (Millipore, US) using an APC-conjugated-OV6 antibody (R&D Systems, Minneapolis) and following manufacturer’s instruction.

### CSCs spheroid formation assay

Cells were magnetically sorted, then 3000 OV6^+^ cells were cultured in Ultra-Low Attachment 6-well plates (Corning Lnc., Coring, NY) for 10–12 days. The tumor spheres was counted under an inversed microscopy and the representative pictures were taken. All experiments were performed in triplicate.

### Quantitative real-time polymerase chain reaction (qRT-PCR)

Total RNA was extracted from cultured ESCC cell lines or magnetic sorted cells using Trizol (Invitrogen, Grand Island, NY) according to the manufacturer’s instruction. The cDNA was synthesized using the PrimeScript RT Reagent Kit (TaKaRa Bio, Shiga, Japan) following the manufacturer’s instructions. Real-time PCR was performed on a Roche Light Cycler 480 (Roche) using SYBR Green PCR Master Mix (TaKaRa Bio, Shiga, Japan). Primer sequences are listed in Table 1. Each measurement was performed in triplicate and the results were normalized by the expression of the GADPH gene. Fold change relative to mean value was determined by 2^-△△Ct^. All experiments were performed in triplicate.

**Table 1:** Sequence if PCR Primers Used in This Study

Gene	Forward primer sequence (5’ −> 3’)	Reverse primer sequence (5’ −> 3’)
CD133	GCCACCGCTCTAGATACTGC	TGTTGTGATGGGCTTGTCAT
CD44	GAGCATCGGATTTGAGA	CATACTGGGAGGTGTTGG
ABCG2	CAGGTTACGTGGTACAAGATGA	GATCAGTGATAAGCTCCATTCC
KLF4	CCATTACCAAGAGCTCATGC	GTGCCTGGTCAGTTCATCTG
SOX2	CAAGATGCACAACTCGGAGA	GCTTAGCCTCGTCGATGAAC
NANOG	CTGCTGGACTGAGCTGGTTGCC	GCTGAGGCCTTCTGCGTCACA
OCT4	AGTGAGAGGCAACCTGGAGA	ACACTCGGACCACATCCTTC
EPCAM	AATCGTCAATGCCAGTGTACTT	TCTCATCGCAGTCAGGATCATAA
CXCR4	ACTACACCGAGGAAATGGGCT	CCCACAATGCCAGTTAAGAAGA
LEF1	AGAACACCCCGATGACGGA	GGCATCATTATGTACCCGGAAT
COMP	GATCACGTTCCTGAAAAACACG	GCTCTCCGTCTGGATGCAG
BMP8A	AGAAAAGCAACGAGCTGCCG	GCCGCGGACGTCATCAAA
ID1	CTGCTCTACGACATGAACGG	GAAGGTCCCTGATGTAGTCGAT
ID3	GAGAGGCACTCAGCTTAGCC	TCCTTTTGTCGTTGGAGATGAC
SMAD9	CTAGGCTGGAAGCAAGGAGAT	GGGGAATCGTGACGCATTT
CHRD	TTCGGCGGGAAGGTCTATG	ACTCTGGTTTGATGTTCTTGCAG
DCN	ATGAAGGCCACTATCATCCTCC	GTCGCGGTCATCAGGAACTT

### Western blot

Whole cultured cells were homogenized in 0.1% SDS and 1 mM PMSF (phenylmethylsulfonyl fluoride) and centrifuged at 12, 000 g for 15 min. Protein extracts were subjected to SDS-PAGE and analyzed using the following primary antibodies: LEF1 (Abcam, ab137872), TGF-β1 (Proteintech,21,898–1-AP), ID1(Santa, sc-133,104), Smad2(CST,5339), Smad3(CST, 9523), p-Smad2(CST, 3108), p-Smad3(CST, 9520) and GADPH (Abcam, ab8245). Then, the membranes were incubated with secondary antibodies (CST,7076,7074) at room temperature for 1 h. The dilution ratio was determined according to the recommended instructions. Protein levels were detected by the Image-Pro Plus 6.0 system (Bio-Rad,1,708,265). Quantification of bands intensity was measured using ImageJ software (version 1.34). All experiments were performed in triplicate.

### Immunohistochemistry (IHC)

The TMAs and 95 ESCC tissues were fixed in 4% methanol, embedded in paraffin, and cut into a thickness of 5 μm. After deparaffinization and rehydration procedures, antigen recovery was performed in a heated citrate buffer (pH 6.0) or EDTA buffer (PH 8.0) for 30 min. Then, the slides were incubated with UltraSensitive Streptavidin Peroxidase Kit (Fuzhou Maixin Biotechnology, Fuzhou, China) and the anti-OV6 (1:50; R&D systems, Minneapolis, MN) and anti-LEF1(1:200, Abcam, ab137876) primary antibodies at 4 °C overnight. Then, diaminobenzidine (DAB) (Dako, Carpinteria, CA, USA) staining was used to image specific markers. The immunostaining staining scores was carried out according to our previous study [11]. Briefly, the immunostaining levels were scored as 0 (negative), 1 + (weakly positive), 2 + (moderately positive), or 3 + (strongly positive). High expression in tumor cells was defined as score ≥ 2 + .

### Chemotherapy drug treatments, soft agar Colony formation assay, and viability assay

ECA109 and TE1 cells from different groups were treated with cis-platinum (2.0 g/mL) for 4 days, and the percentage of OV6^+^ cells was then measured by flow cytometry. For the soft agar assay, magnetically sorted OV6^+^ cells were cultured in 1 mL of 0.7% agarose with DMEM-mixed upper gel on 6-well plates. Then, 0.6 g/mL of cis-platinum was added into 6-well plates and incubated for 2 weeks. Colony formation numbers was determined by microscope counting. Cell viability after drug treatment was assessed by a cholecystokinin-8 assay (CCK8).

### Xenograft mice

Six-week-old male BALB/c nude mice were purchased from Shanghai Experimental Center (Shanghai, China). Mice were used to evaluate the effects of LEF1 upregulation and downregulation of on tumorigenicity and tumour growth. Briefly, different numbers of LV-LEF1, LV-GFP, and LV-shLEF1 ECA109 cells were suspended in 200 μL of DMEM and Matrigel (1:1) (Corning) and injected into the subcutaneous tissue of mice. Tumor size and incidence were measured weekly. Mice were sacrificed at the indicated time points according to the protocols approved by the SMMU Animal Care Facility and the National Institutes of Health guidelines. Tumors were harvested for assessment of tumour size, tumour incidence, western blot and immunohistochemistry analysis.

### RNA-seq

RNA-seq was performed on three biological replicates. LEF1-overexpression ECA109 cells and negative control ECA109 cells were used for RNA-seq analysis by Shanghai Novelbio corporation (Shanghai, China). Total RNA from ECA109 LV-GFP/LV-LEF1 was extracted by Trizol and kept at − 80 °C. DNA was removed using a DNA-free DNA Removal Kit (Thermo Fisher, AM1906). The RNA quantitation and quality measurement were performed using a Bioanalyzer 2200 (Agilent Technologies, USA). RNA with a RIN (RNA integrity number) > 8.0 was considered acceptable for cDNA library construction. Differentially expression genes were considered to be significant between groups when the *P*-value was < 0.05 and the fold change of expression was ≥1.50-fold or ≤ 0.67-fold. All RNA-Seq files are available from the GEO database (accession number: GSE128914).

### Luciferase report assay

Different groups of ESCC cell were cultured in 24-well plates in triplicate. After 24 h, ESCC cells were transfected with the indicated plasmids and pRL-TK *Renilla* plasmid using lipofectamine 2000 reagent (Thermo Fisher, USA, No.11668019). Luciferase and *Renilla* signals were measured 48 h after transfection by a Dual-Luciferase Reporter Assay Kit (Promega, No. E1980). Data were normalized by the division of firefly luciferase activity with that of *Renilla* luciferase to eliminate transfection efficiency difference.

### Chromatin immunoprecipitation (ChIP) assays

We identified the LEF1-bingding sites on ID1 promoter region by using JASPAR and also referred to Chip-Seq data of LEF1 on GEO. ChIP assay was conducted with SimpleChIP® Enzymatic Chromatin IP Kit (CST, 9003) following the manufacturer’s instructions. Briefly, ECA109 and TE1 cells (4 × 10^6^) were cross-linked by using 1% formaldehyde and used for each immunoprecipitation experiment. Chromatin was digested with the micrococcal nuclease. 2% aliquots of lysates were used as an input reference. LEF1 antibody (Abcam, ab137872) or normal rabbit IgG (CST, 2729) were incubated with the other immunoprecipitation samples at 4 °C for overnight. Then, the crosslink DNA was reversed by NaCl and proteinase K. Immunoprecipitated DNA was amplified by PCR using their specific primers. The primer sequences for ID1 gene were 5′-CGCCCGCTTTAAATTTCGG-3′ (forward), and 5′- CACAGATGAGAGAAA.

TTGAGGC − 3′ (reverse). The signals were calculated as the percentage of input.

### Statistical analysis

SPSS 22 software (SPSS, Chicago, IL, USA) was used to statistically analyse the data. The association between markers and clinical features were analysed by chi-square test, Fisher’s exact test or two-side t-test. Spearman’s rank correlation was used to analyse the association between LEF1 and OV6 expression. Survival curves were analysed by using the Kaplan-Meier method. Multivariate analysis of survival was examined by Cox proportional hazard regression model. The experimental data were obtained in three independent experiments and analysed by ANOVA. *P* < 0.05 indicated statistical significance.

## Results

### Expression of LEF1 in primary ESCC tissues

**Fig. 1:**
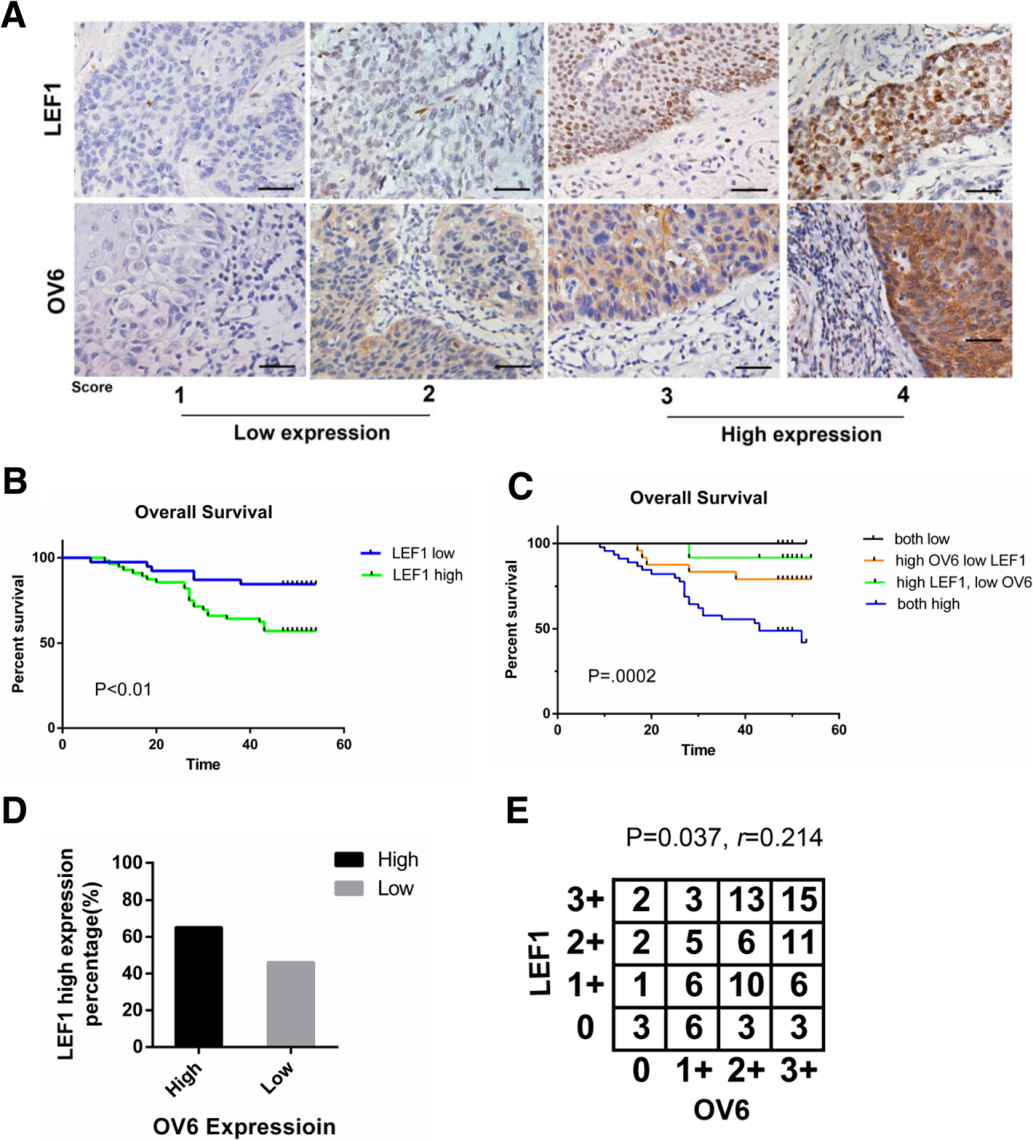
LEF1 and OV6 expression in ESCC tissues and its clinicopathological characteristics. **a** Immunochemistry analysis on expression of LEF1 and OV6. Representative staining intensity of LEF1 and OV6 represent different expression levels (scale bar = 100 μm). **b** Overall survival rate for patients with low LEF1 expression (blue line) and patients with high LEF1 expression (green line), *P* < 0.01. **c** The overall survival rates of 95 patients with ESCC were compared with different groups by Kaplan–Meier analysis, *P* = 0.0002. **d** The percentage of patients with high LEF1 staining was higher in the OV6-high group. **e** Correlation between expression levels of OCT4 and LEF1 in ESCC measured by spearman’s rank correlation analysis, Spearman r = 0.214, *P* = 0.037

Our previous studies reported that LEF1 is predominantly expressed in ESCC cell lines and tumor tissues and that the positive expression of LEF1 is correlated with aberrant clinicopathological characteristics of ESCC patients [18]. LEF1 expression varied in different ESCC specimens, which were scored as 0,1,2, or 3 according to the intensity of LEF1 staining. We then investigated the relationship between LEF1 expression and the clinicopathological features of ESCC patients. Based on LEF1 expression levels in ESCC tissues, all 95 patients (Fig. 1a) and additional 243 patients (Additional file 1: Figure S1A) were classified into 2 groups: low expression (scored as 0 and 1) and high expression (scored as 2 and 3). As shown in Table 2, Additional file 2: Table S1 and Additional file 2: Table S2, the LEF1 high expression group was significantly associated with multiple malignant clinicopathological features. More importantly, the difference in overall survival was also significant between the two groups. A Kaplan-Meier curve unveiled that patients with high expression levels of LEF1 were associated with a lower overall survival rate (Fig. 1b, Additional file 1: Figure S1B). Furthermore, we performed multivariate survival analysis by using the Cox multivariate regression model. The results revealed that LEF1 was an independent factor affecting the overall survival rate of patients with ESCC (Table 3).

**Table 2:** Correlation between LEF1 expression and clinicopathological characteristics in 95 patients

	LEF1		
	LEF1 high (57) LEF1 low (38) *P* value^a^		
Sex			
Male	48	30	0.598
Female	9	8	
Age (year)^b^			
> 65	16	9	0.812
≤ 65	41	29	
T_grade			
1	11	17	**0.04**
2	16	9	
3	29	11	
4	1	1	
Lymph node metastasis			
N0	29	28	0.174
N1	15	6	
N2	10	3	
N3	3	1	
TNM stage			
I	10	21	**< 0.01**
II	22	9	
III	25	8	
Death			
yes	25	5	**< 0.01**
no	32	33	

^a^Stactical signaficance was figrued out by chi-square test or Fisher’s exact test

^b^Data are presented as mean ± S.D

**Table 3:** Multivariate analysis of Cox proportional hazards model for esophageal squamous cell carcinoma cases

							95% CI for Exp(B)	
	B	SE	Wald	df	Sig.	Exp(B)	Lower	Upper
Age	.014	.029	.225	1	.635	1.014	.985	1.073
Gender	.173	.460	.142	1	.707	1.189	.482	2.932
T grade	.066	.431	.024	1	.878	1.069	.459	2.486
Lymph node metastasis	.363	.216	2.814	1	.093	1.437	.941	2.195
TNM stage	.354	.328	1.169	1	.280	1.425	.750	2.708
LEF1 Level	−1.116	.508	4.824	1	**.028**	.327	.121	.887

B, partial regression coeffcients; Exp(B), hazard ratio; SE, standard error; 95.0% CI for Exp(B): 95.0% confdence interval for Exp(B); Wald:Wald chi-square

### Correlation between LEF1 and OV6 in ESCC tissues

Our previous study reported that OV6 was a possible CSC marker for ESCC and was associated with poor prognosis in ESCC patients [11]. To further study the relationship between OV6 and LEF1, we evaluated the OV6 and LEF1 expression levels in the same samples from 95 ESCC patients. Based on their tumor LEF1 and OV6 content, the patients were classified into 4 groups: group A, high OV6 and LEF1 intensity; group B, high OV6 but low LEF1 intensity; group C, high LEF1 but low OV6 intensity; and group D, low OV6 and LEF1 intensity (Fig. 1a). In ESCC patients expressing high levels of OV6, the percentage of patients with a high level of LEF1 staining was 65.2%, which was higher than that of patients expressing low levels of OV6 (Fig. 1d). Moreover, compared with the other group, the group A patients were more likely to exhibit aberrant clinicopathological features (Table 4). Furthermore, we tested the correlation between OV6 and LEF1 expression by Spearman’s rank correlation (Fig. 1e). The results indicated a significant association between the expression of OV6 and LEF1 in ESCC.

**Table 4:** Clinicopathologic characteristic by OV6 and LEF1 expression

OV6/LEF1 expression					
	Both high (45)	High-OV6, Low-LEF1(24)	High-LEF1 Low-OV6(12)	Both Low (14)	*P* value^a^
Sex					
Male	39	20	9	10	0.477
Female	6	4	3	4	
Age (year)^b^					
> 65	13	5	3	4	0.93
≤ 65	32	19	9	10	
T_grade					
1	8	10	3	7	**0.017**
2	10	8	6	1	
3	26	5	3	6	
4	1	1	0	0	
Lymph_node_metastasis					
N0	22	16	7	12	0.51
N1	11	5	4	1	
N2	9	2	1	1	
N3	3	1	0	0	
TNM stage					
I	7	13	4	8	**0.011**
II	17	5	5	4	
III	21	6	3	2	
Death					
yes	23	5	1	0	**< 0.01**
no	22	19	11	14	

^a^Statistical significance was figured out by chi-square test or Fisher’s exact test for categorical/binary measures and ANOVA for continuous measures

^a^Data are presented as mean ± S.D

Based on the association between OV6 and LEF1, we analyzed their impact on ESCC patient survival. Follow-up data from 95 patients were analyzed using the Kaplan-Meier method to evaluate survival curves. The results indicated that patients with high expression of both OV6 and LEF1 were significantly associated with a lower overall survival rate (Fig. 1c). Therefore, the combination of OV6 and LEF1 expression can be a prognostic predictor for ESCC.

### Upregulation of LEF1 promoted a CSC-like phenotype in human ESCC cells in vitro

**Fig. 2:**
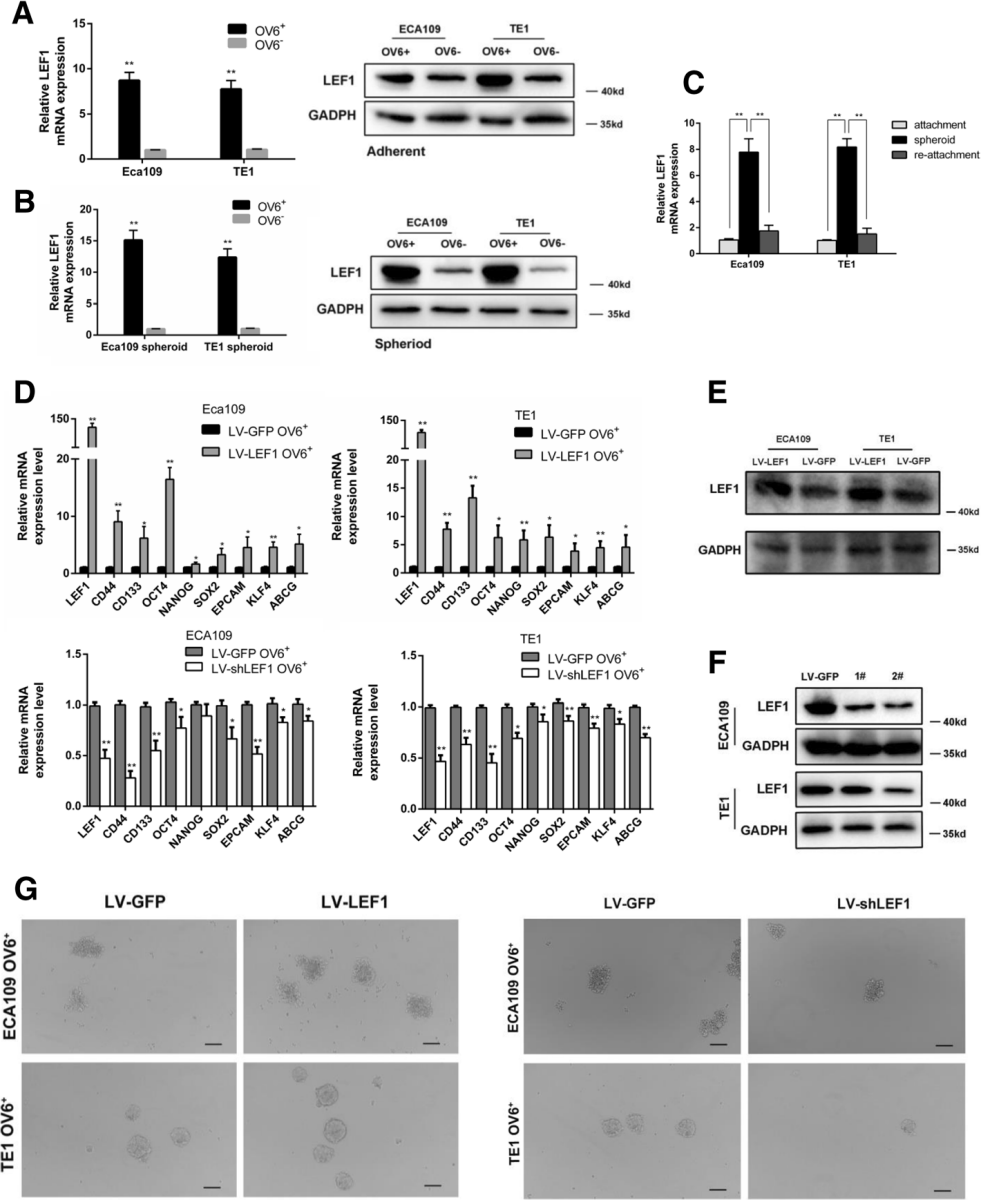
LEF1 is predominantly expressed in esophageal CSCs and promotes a CSC-like phenotype in ESCC cells in vitro. **a**, **b** qRT-PCR and western blotting analysis of magnetically sorted OV6^+^ adherent (**a**) and spheroid(**b**) subpopulations was performed to evaluate the relative mRNA and protein expression levels, respectively, of LEF1 in ECA109 and TE1 cells. Data are shown as the mean ± SD, *P < 0.05, **P < 0.01. **c** qRT-PCR analysis of LEF1 expression of ECA109 and TE1 cells in different culture conditions. Data are shown as the mean ± SD, **P < 0.01. **d** qRT-PCR analysis was performed for LEF1 and the stem cell-associated genes in magnetically sorted LV-LEF1 OV6^+^ or LV-shLEF1 OV6^+^ cells of two ESCC cell lines. Data are shown as the mean ± SD, *P < 0.05, **P < 0.01. **e**, **f** The protein level of LEF1 in the LV-LEF1 group and LV-shLEF1 group was shown in ECA109 and TE1 cells. **g** Tumor spheroid formation assay indicated that LV-LEF1 OV6^+^ cells were able to generate an increased number and size of primary and secondary spheroids in ECA109 and TE1 cell lines, whereas LEF1-silenced OV6^+^ cells exhibited the opposite effect (scale bar = 100um). Data are shown as the mean ± SD *P < 0.05, **P < 0.01. All experiments were performed in triplicate

**Fig. 3:**
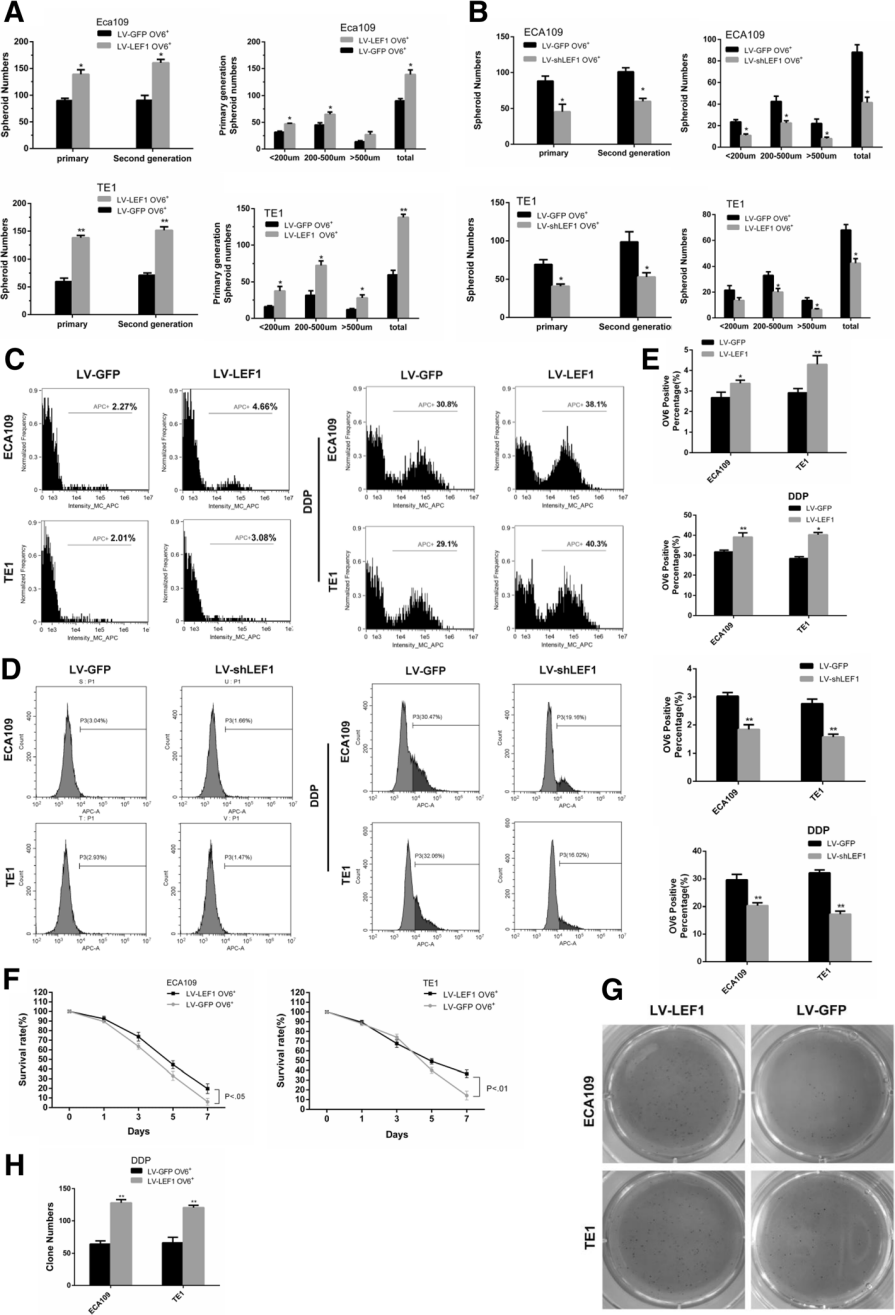
Overexpression of LEF1 promotes chemoresistance of esophageal CSCs. **a**, **b** Representative quantification of tumor sphere formation by LEF1-overexpressing cells (**a**) or LEF1-silenced cells (**b**). Bars represent the mean ± SD of three independent experiments, *P < 0.05, **P < 0.01. **c**, **d** Flow cytometric analysis showed that the percentage of OV6^+^ cells was significantly increased in LV-LEF1 cells and that the standard 4 days of chemotherapy with cis-platinum (2.5 g/ml) augmented the percentage of OV6^+^ subpopulation (**c**), whereas LEF1-silenced cells exhibited the opposite effect (**d**). Data are shown as the mean ± SD, *P < 0.05, **P < 0.01. **e** Representative quantification of the percentage of OV6^+^ cells by LEF1-overexpressing cells (upper panel) or LEF1-silenced cells (lower panel). **f** Cyclocystokinin-8 assay (CCK8) indicated that LEF1 overexpression enhanced the cell viability of ECA109 and TE1 OV6^+^ cells in the presence of cis-platinum (0.6 g/ml). Data are shown as the mean ± SD, P < 0.01. **g**, **h** Increased ability of colony formation was also observed in LV-LEF1 groups compared with LV-GFP groups after treated with cis-platinum (0.6 g/ml) for 14 days. Data are presented as the mean ± SD *P < 0.05, **P < 0.01. All experiments were performed in triplicates

Our previous study indicated that OV6^+^ cells could represent a potential CSC population in ESCC [11]. Here, we showed that the expression of LEF1 mRNA and protein levels were significantly increased in the magnetically sorted OV6^+^ subpopulation of adherent ECA109 and TE1 cells (Fig. 2a). Likewise, increased amounts of LEF1 were also found in the OV6^+^ subpopulation of spheroidal ECA109 and TE1 cells (Fig. 2b). Interestingly, when we seeded these spheroidal cells back into adherent culture conditions, the increased level of LEF1 expression returned (Fig. 2c). Collectively, these data indicated that LEF1 is highly expressed in the CSCs of ESCC. To further explore the role of LEF1 in the CSC-like phenotype, we applied a lentivirus-based approach to stably express LEF1(LV-LEF1) or shLEF1(LV-shLEF1) in EAC109 and TE1 cells, and the expression of LEF1 was tested by western blot (Fig. 2e, f). qRT-PCR analysis revealed that the mRNA levels of CSC markers were significantly elevated in OV6^+^ LV-LEF1 cells and conversely downregulated in OV6^+^ LV-shLEF1 cells compared with those in control OV6^+^ LV-GFP cells (Fig. 2d). Moreover, flow cytometric analysis also indicated that the overexpression of LEF1 led to an increase in the number of OV6^+^ ECA109 and TE1 cells, while the downregulation of LEF1 decreased the percentage of OV6^+^ cells (Fig. 3c left panel, 3D left panel, 3E). Next, a tumor sphere formation assay was used to explore the effect of LEF1 on CSC self-renewal properties in ESCC. As shown in Figs. 2g and 3a, b, LEF1-transduced OV6^+^ cells formed a larger number of spheroidal cells with more varied sizes than the OV6^+^ LV-GFP cells, whereas LEF1-silenced OV6^+^ cells exhibited the opposite effect on spheroidal cell formation.

A strong resistance to chemotherapy is one of the most important properties of CSCs. The upregulation of LEF1 increased the percentage of OV6^+^ ECA109/TE1 cells in the total population following cis-platinum treatment for 4 days (Fig. 3c right panel, 3D right panel, 3E). In addition, the overexpression of LEF1 enhanced the colony formation capability (Fig. 3g, h) and cell viability (Fig. 3f) of ECA109 and TE1 OV6^+^ cells in the presence of cis-platinum. Taken together, these data suggest that the overexpression of LEF1 enhances the CSC-like phenotype in ESCC cells in vitro.

### The overexpression of LEF1 promotes Tumorgenicity in ESCC in vivo

**Fig. 4:**
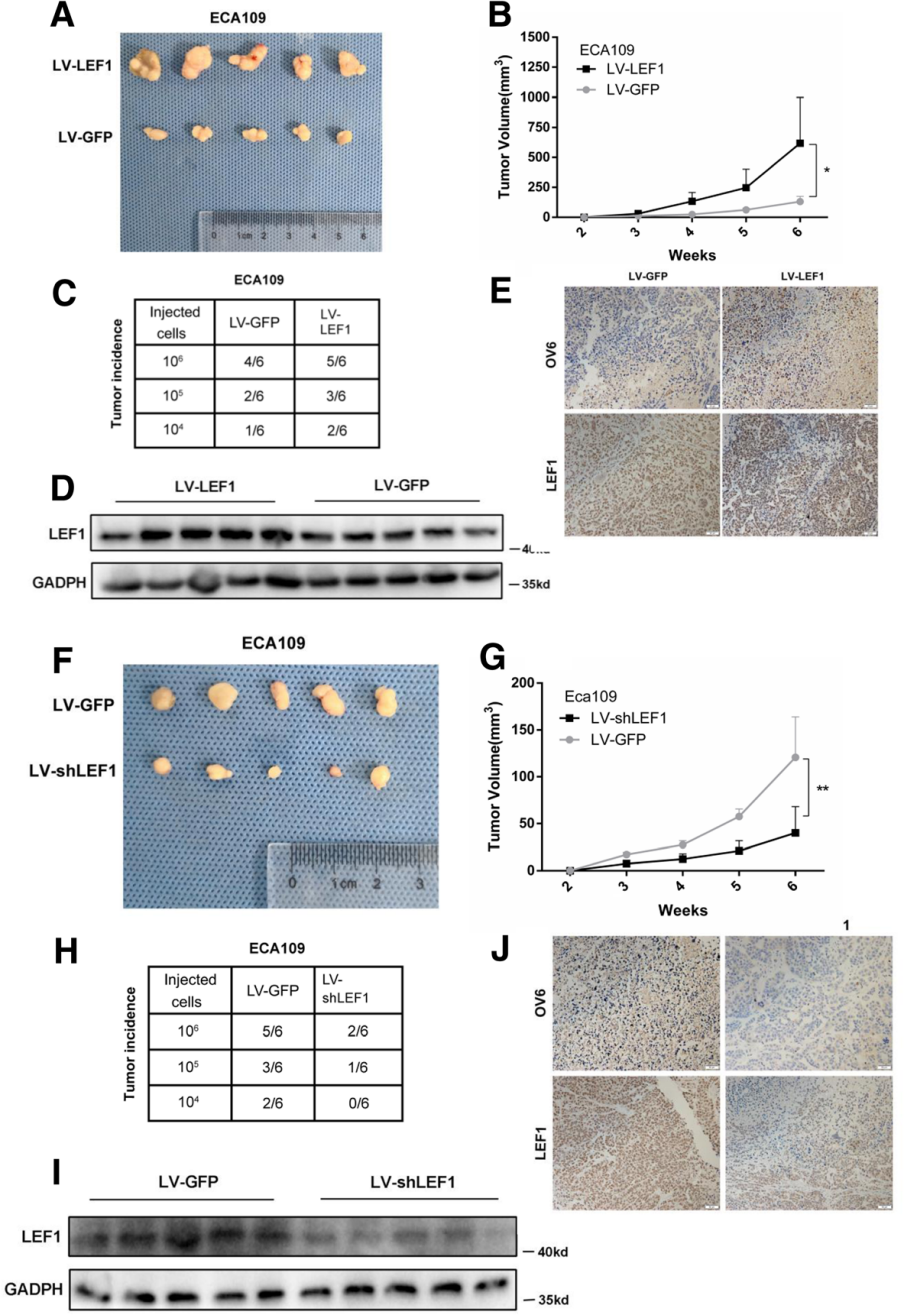
Overexpression of LEF1 enhances the tumorigenicity of ESCC cells in vivo. **a**, **b** Representative tumors from mice injected with 10^6^ LV-LEF1 or LV-GFP ECA109 cells after 6 weeks were presented. Tumor volumes from each group were measured weekly. **c** Mice were injected with the indicated number of LV-LEF1 or LV-GFP ECA109 cells and tumor incidence in the mouse xenografts were obtained after 28 days. **d**, **e** Western blot (**d**) and Immunohistochemistry (**e**) analysis the LEF1 and OV6 expression on mouse subcutaneous tumors inoculated after 6 weeks. Scale bar = 50 μm. **f**-**j** Indicated number of LV-shLEF1 and LV-GFP ECA109 cells were injected into mice. Tumor volumes (**f**, **g**), tumor incidence (**h**), expression level of LEF1 and OV6 (**i**, **j**) were presented. Scale bar = 50 μm

To further demonstrate the effect of LEF1 in mediating the CSC-like phenotype in ESCC cells in vivo, we injected LV-LEF1 and LV-GFP ECA109 cells with Matrigel into the subcutaneous tissues of BALB/c nude mice. As shown in Fig. 4a, b, the overexpression of LEF1 significantly increased tumor growth. Furthermore, a difference in tumor incidence was also observed between these two subpopulations after the implantation of 10^4^, 10^5^, and 10^6^ cells (Fig. 4c). Consistent with this, increased OV6 and LEF1 expression levels were also observed in the LV-LEF1 group (Fig. 4d, e). Conversely, the downregulation of LEF1 significantly decreased tumor growth (Fig. 4f, g) and tumorigenicity (Fig. 4h) and caused a decrease in OV6 and LEF1 expression levels compared with the vector control (Fig. 4i, j). Therefore, these results indicate that LEF1 promotes the tumorigenicity of ESCC cells in vivo.

### The TGF-β signaling pathway contributes to the LEF1-mediated CSC-like phenotype in ESCC cells

**Fig. 5:**
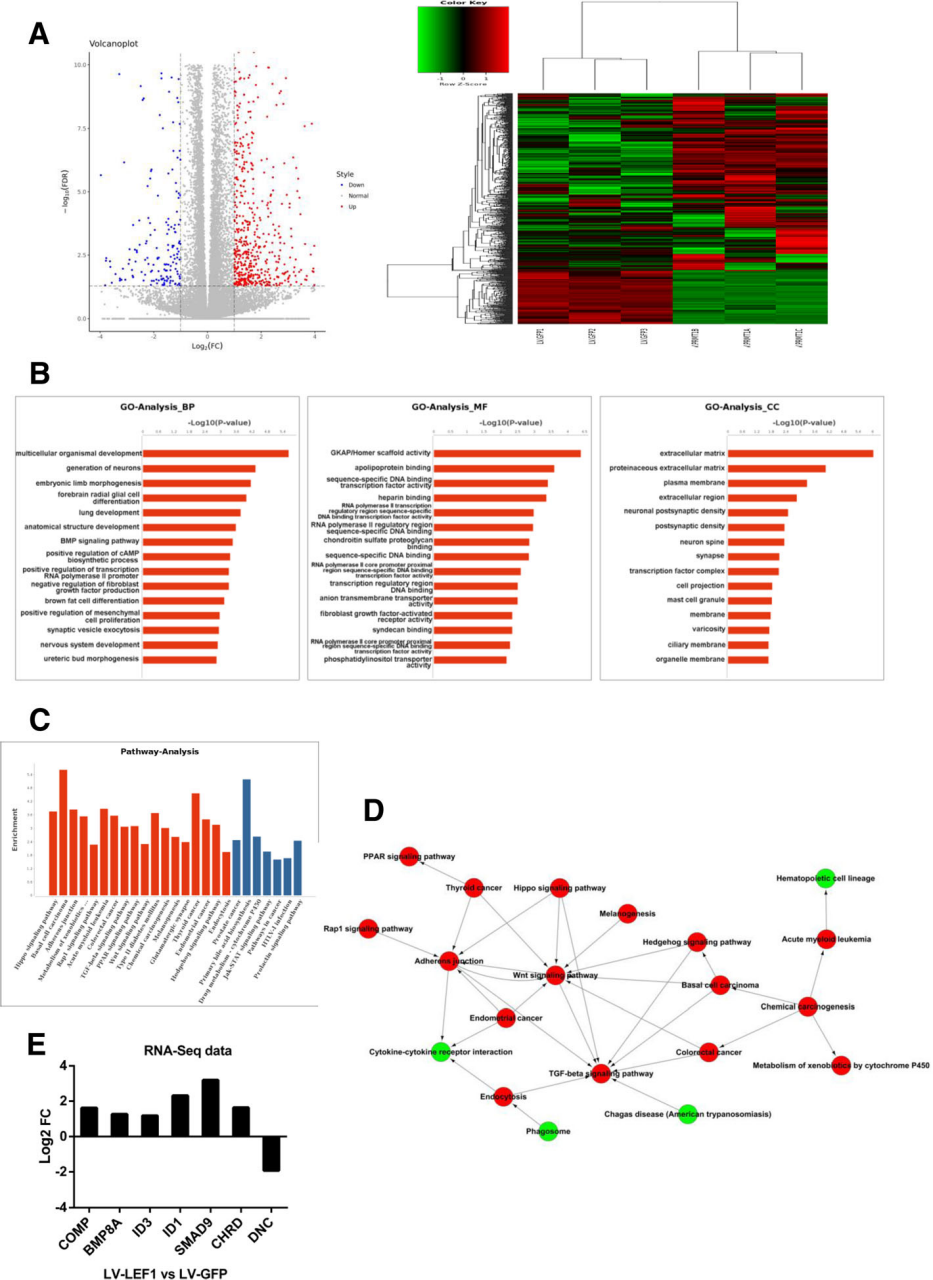
Overexpression of LEF1 promotes TGF-β signaling pathway. **a** Heatmap and volcano plot of differentially expressed transcripts. **b**, **c** Gene ontology (GO) analysis (**b**) and pathway analysis (**c**) based on all identified transcripts. *P*-value of GO analysis is listed for each category. P-value of pathway is colored in red (P < 0.05) and blue (0.05 < *P* < 0.1). **d** Pathway-act network of up-regulated (red) or down-regulated (green) pathways according to pathway database. **e** Gene expression fold change of TGF-β pathway from RNA-Seq data

To investigate the mechanism related to the LEF1-mediated CSC-like phenotype in ESCC, we applied RNA-Seq analysis to profile the transcriptome in LEF1-overexpressing ECA109 cells versus control ECA109 cells. A total of 883 genes were identified as differentially expressed and were analyzed to characterize their potential biological processes, molecular functions, pathways, and pathway-act network (Fig. 5a-d). Among the pathways known to regulate the self-renewal properties of cancer cells, the TGF-β signaling pathway was significantly dysregulated in LEF1-overexpressing ECA109 cells (Fig. 5c). Moreover, gene ontology (GO) analysis revealed that the overexpression of LEF1 significantly affected the positive regulation of mesenchymal cell proliferation, which might be the reason for the development of cancer stem cells. Pathway-act network also indicated that the TGF-β signaling pathway was located in the core area of the pathway map and was the downstream target of Wnt signaling pathway.

**Fig. 6:**
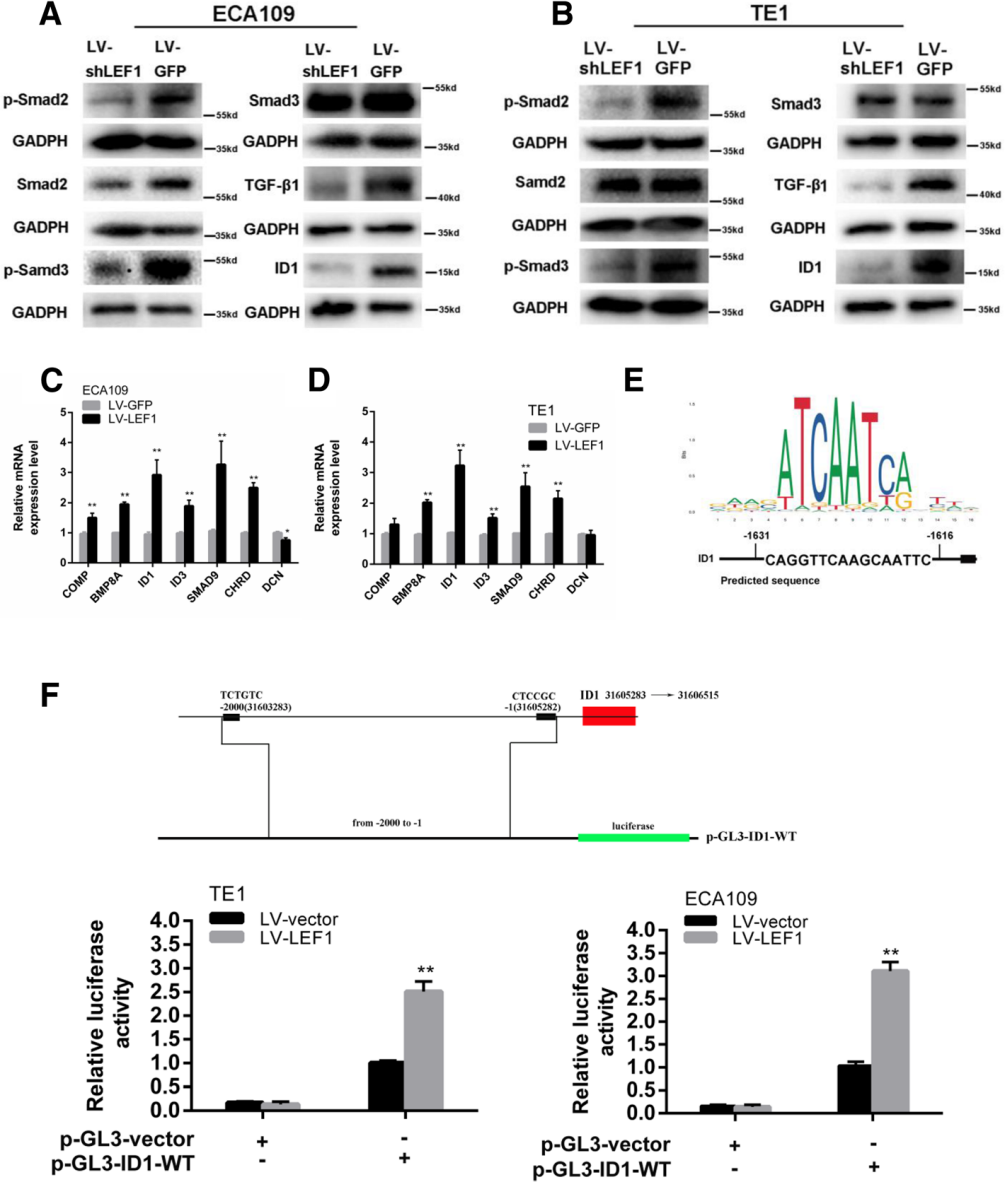
LEF1 activates TGF-β signaling and directly upregulates ID1 in HCC cells. **a**, **b** Western blot of GADPH (loading control), phosphorylated and total protein of Smad2 and Smad3, TGF-β1, ID1 in ECA109 and TE1 with LEF1 knockdown by shRNA. **c**, **d** Seven differentially expressed transcripts were validated by qRT-PCR in LEF11 overexpression ECA109 and TE1 cells. Data is shown as the mean ± SD, *P < 0.05, **P < 0.01. **e** The sequence logo of a potential LEF1 binding site in JASPAR and potential binding site in the ID1 sequence. **f** Construction of plasmid and overexpression of LEF1 significantly enhanced the relative luciferase activity compared with control group in ECA109 and TE1 cells, *P < 0.05, **P < 0.01. All experiments were performed in triplicate

To explore the effect of LEF1 on the TGF-β signaling pathway, we tested the expression levels of active Smad2, Smad3, p-Smad2, p-Smad3, and TGF-β by western blot analysis. As shown in Fig. 6a, b, the overexpression of LEF1 increased the levels of p-Smad2, p-Smad3, and TGF-β in both ECA109 and TE1 cells. Next, we examined the RNA-Seq data and found that seven significantly differentially expressed genes were enriched in the TGF-β pathway, including DCN, COMP, BMP8A, ID3, ID1, SMAD9, and CHRD (Fig. 5e). qRT-PCR analysis also validated the expression of these genes, which was consistent with the results from our transcriptome analysis (Fig. 6c, d).

A previous study suggested that the TGF-β/ID1 signaling pathway contributes to the stem-like phenotype and promotes the metastatic colonization of breast cancer cells [30]. According to the RNA-Seq and qRT-PCR data, we performed JASPAR database analysis to further verify the downstream targets of LEF1 in the TGF-β signaling pathway, which predicted that the *ID1* gene promoters contain LEF1 binding sites (Fig. 6e). A luciferase reporter assay confirmed that LEF1 could directly bind to and positively regulate *ID1* (Fig. 6f). Moreover, Chip-qPCR assay also showed that LEF1 directly bind to the promoter of ID1 in ECA109 and TE1 cells (Additional file 1: Figure S1C, S1D). Consistent with this, western blot analysis indicated that the overexpression of LEF1 increased the ID1 protein levels in ESCC cells (Fig. 6a, b). Therefore, ID1 has been shown to be a related target protein of LEF1 downstream of the TGF-β signaling pathway.

### ID1 is positively correlated with LEF1 expression and poor patient prognosis in human esophageal squamous cell carcinoma

**Fig. 7:**
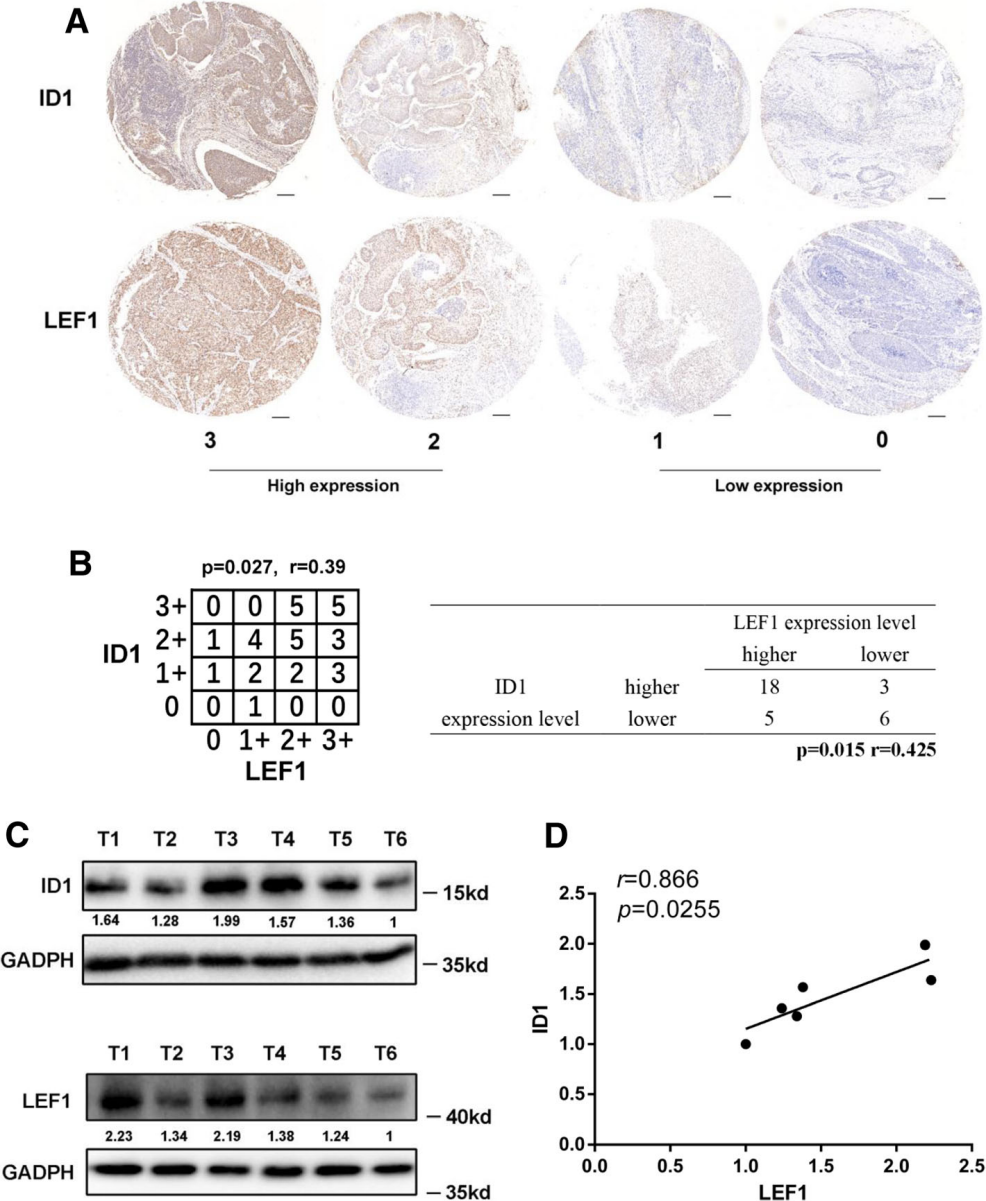
The association of LEF1 with ID1 expression in human ESCC specimen. **a** Representative images of LEF1 and ID1 staining by IHC in ESCC TMA sections of 31 patients (scale bar, 50 μm). The immunostaining levels of LEF1 and ID1 were also scored from 0 to 3. **b** Correlation of expression levels of LEF1 and ID1 is shown. **c**, **d** Western blotting analysis (**c**) and correlation analyses (**d**) of LEF1 expression with expression of ID1 in six freshly collected human ESCC samples

To confirm the correlation between the expression levels of LEF1 and ID1, tissue microarray (TMA) analysis by immunohistochemical staining was performed in a total of 129 clinical ESCC specimens (Fig. 7a, Additional file 1: Figure S1A upper panel). ESCC patients exhibiting a high level of LEF1 expression were divided into a low ID1 expression group and a high ID1 expression group. Moreover, the expression of LEF1 was also positively correlated with ID1 expression in TMA samples (Fig. 7b, Additional file 1: Figure S1E). In addition, we validated the correlation between LEF1 and ID1 expression in seven freshly collected ESCC tissue specimens. The results indicated that LEF1 expression was positively correlated with the expression of ID1(Fig. 7c, d). Taken together, these results indicate that the overexpression of LEF1 is positively associated with the expression of ID1, which in turn activates the TGF-β pathway and promotes the tumorigenicity of ESCC.

## Discussion

Esophageal cancer (EC) is one of the most malignant tumors worldwide and has a high mortality rate [31]. Most esophageal cancers are histologically classified as esophageal squamous cell carcinoma (ESCC), which accounts for approximately 80% of all EC [32]. Despite advances in surgery, chemotherapy, and radiotherapy in esophageal squamous cell carcinoma (ESCC) treatment, the prognosis of ESCC patients is still poor [33].

Cancer stem cells (CSCs) are correlated with cancer recurrence and metastasis due to their self-renewal properties and capacity for drug resistance [34]. It was recently reported that CSCs directly contribute to therapy resistance in and tumor progression of ESCC [35]. Recent studies have verified that OV6, an epithelial marker, can be used as a CSC marker in many cancers such as hepatocellular carcinoma and rectal cancer [36]. The present study found that OV6 was also upregulated in ESCC, and the high expression of OV6 was closely associated with the clinicopathological characteristics and prognosis of ESCC patients [11]. The elimination of CSCs might prevent cancer progression and recurrence in patients. Compared with traditional treatment, targeted therapy has shown a unique advantage. Therefore, it is imperative to identify novel cancer targets and investigate their clinical relevance to targeted treatment.

LEF1 is a transcription factor that primarily participates in the Wnt/β-catenin signaling pathway. LEF1 is also a facilitator of epithelial-mesenchymal transition (EMT), a feature of cancer cell migration and invasion, as well as cancer cell proliferation and viability [37]. A previous study suggested that LEF1, as a candidate CSC marker, was highly elevated during EMT in hepatocellular carcinoma [38]. Moreover, our previous study also reported that LEF1 was highly expressed in esophageal squamous cell carcinoma and was closely related to tumor progression and poor patient prognosis [18]. In colorectal cancer patients, the overexpression of LEF1 represented a risk factor for the poor overall survival of CRC patients, and increased expression of LEF1 with decreased expression of Notch2 could be used to facilitate the early detection of colorectal cancer [39]. We speculated that LEF1 8could modulate gene transcription and tumorigenesis independently by processing the Wnt/β-catenin signaling pathway, which might contribute to maintaining CSC-like characteristics. In this study, we found that the overexpression of LEF1 promoted the stem cell-like properties of CSCs in ESCC, including spherical tumor formation, chemoresistance, and tumorigenicity. Moreover, the combination of high levels of LEF1 and OV6 predicted the aberrant clinicopathological characteristics and poor patient prognosis in ESCC patients. These results indicate the crucial role of LEF1 in the regulation of the CSC-like phenotype in ESCC cells, which might hold promise for the development of novel therapeutic targets for tumor treatment.

The TGF-β signaling pathway is an important effector of a number of pathways that plays complex roles in the development, progression, and metastatic potential of cancers and is correlated with tumor invasion and poor patient prognosis [40]. Recently, many studies have indicated an important role for the TGF-β signaling pathway in promoting EMT and a CSC-like phenotype [41]. Moreover, EMT, a key process that is often activated during cancer invasion, has been reported to generate cells with stem cell-like properties [41]. To investigate the mechanism of LEF1’s effect on the tumorigenesis and self-renewal properties of ESCC cells, we applied the RNA-Seq method and found that the TGF-β signaling pathway is significantly activated after LEF1 overexpression. Then, we selected a series of upregulated genes (DCN, COMP, BMP8A, ID3, ID1, SMAD9, and CHRD) from the RNA-Seq data that are related to the TGF-β pathway and validated their expression by qRT-PCR analysis. Based on a luciferase reporter assay, we confirmed that LEF1 directly binds to the promoter of the ID1 gene, which was shown to regulate the CSC-like phenotype in many tumors [30]. In addition, a significant correlation between LEF1 and ID1 expression was observed in clinical ESCC patients, strongly suggesting that LEF1 regulation of the CSC phenotype is associated with ID1 expression.

In summary, we demonstrated that the overexpression of LEF1 was closely associated with aberrant clinicopathological characteristics and might be used as an independent prognostic factor of ESCC. The association of LEF1 with OV6 expression in ESCC has been statistically proven, and the concomitant elevated expression of LEF1 and OV6 might contribute to poor overall survival rates in ESCC patients. Moreover, the overexpression of LEF1 directly upregulates *ID1* and activates the TGF-β signaling pathway, thereby promoting tumorigenicity and the CSC-like phenotype. In-depth investigation of the function of TFAP4 in ESCC would partially shed light on the mechanisms underlying the high rate of recurrence in ESCC and provide a potential therapeutic target to prevent the recurrence of ESCC.

## Conclusions

In the present manuscript, we showed the elevated expression of LEF1 was associated with aberrant clinicopathological characteristics and poor patient prognosis of ESCC patients. We also investigated the clinicopathological significance of the association of OV6 with LEF1 expression. Furthermore, we identified LEF1 as a key regulator of CSC-like phenotype and was responsible for ESCC tumorigenesis. Mechanically, LEF1 overexpression in ESCC directly upregulates ID1 and activates TGF-β pathway.

This work underlines the importance of LEF1 in regulating CSC-like phenotype and proposes potential new therapeutic target to better treat this deadly disease.

## Additional files

Additional file 1: **Figure S1.** LEF1 and ID1 expression in ESCC samples and the transcription of ID1 is regulated by LEF1. (A) LEF1 and ID1 staining by IHC in ESCC TMAs were showed. Upper panel was used for analysis of expression association between LEF1 and ID1.A total of 243 LEF1 staining patients were used for clinicopathological characteristics analysis. (B) The overall survival rates of 243(left panel) and 338 patients (right panel) with ESCC were compared with different groups by Kaplan–Meier analysis. (C) Diagram showed that LEF1 bound to the promoter of ID1 and putative LEF1 binding sites are indicated. (D) ChIP assays confirmed that LEF1 could bind to the ID1 promoter in ECA109 and TE1 cells. Quantification of immunoprecipitated DNA was shown by qRT-PCR. (E) Correlation of expression levels of LEF1 and ID1 in 98 patients (left panel) and total 129 patients (right panel) are shown. (PDF 238 kb)

Additional file 2: **Table S1.** Correlation between LEF1 expression and clinicopathological characteristics in 243 patients. **Table S2.** Correlation between LEF1 expression and clinicopathological characteristics in total 338 patients. (DOCX 21 kb)

## Data Availability

All data generated or analyzed during this study are included in this published article.

## Abbreviations

CSC: Cancer stem cell
DMEM: Dulbecco’s modified Eagle’s medium
ESCC: Esophageal squamous cell carcinoma
LEF1: Lymphoid enhancer-binding factor 1
LV: Lentiviral
OV6: anti-OV6 antibody
qRT-PCR: Quantitative Real-Time Polymerase Chain Reaction
