# Supplementary figures and images for: The transcription factor LEF1 promotes tumorigenicity and activates the TGF-β signaling pathway in esophageal squamous cell carcinoma
Source: J Exp Clin Cancer Res. 2019 Jul 11;38:304. doi: 10.1186/s13046-019-1296-7 (PMC6625065; doi:10.1186/s13046-019-1296-7)

Supplement Fig.1

A

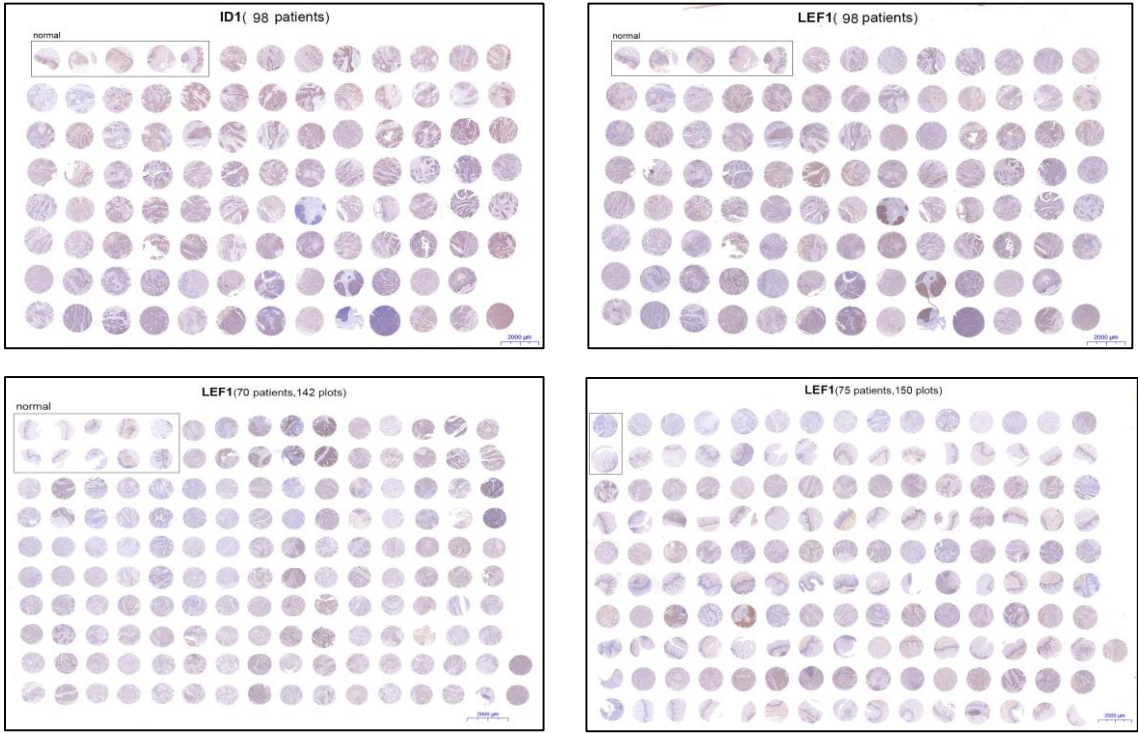

B

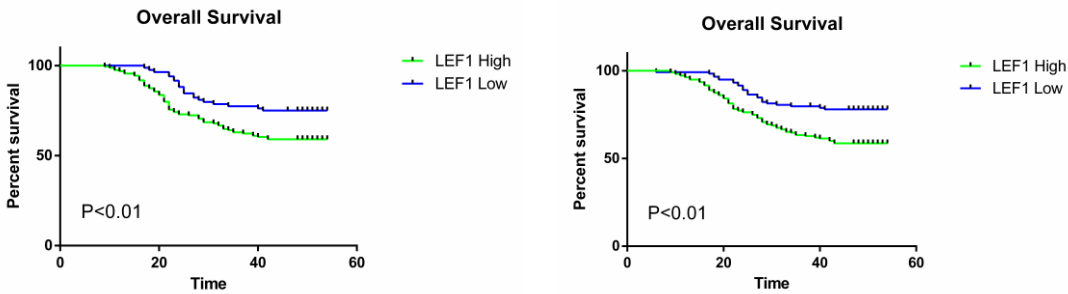

C

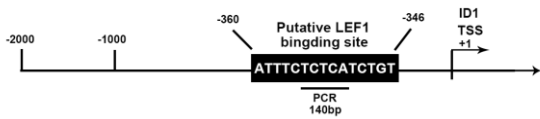

D

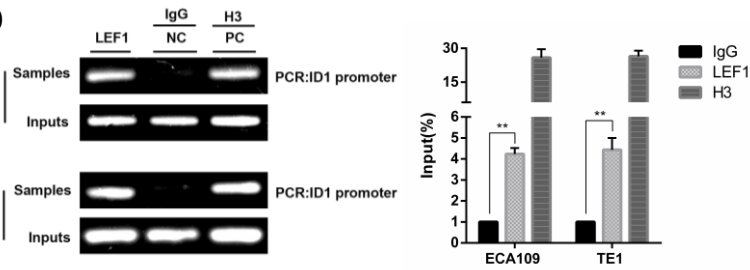

E

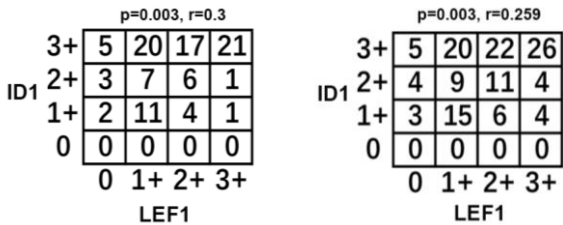

Supplement: Supplementary file 1 — Figure S1. LEF1 and ID1 expression in ESCC samples and the transcription of ID1 is regulated by LEF1. (A) LEF1 and ID1 staining by IHC in ESCC TMAs were showed. Upper panel was used for analysis of expression association between LEF1 and ID1.A total of 243 LEF1 staining patients were used for clinicopathological characteristics analysis. (B) The overall survival rates of 243(left panel) and 338 patients (right panel) with ESCC were compared with different groups by Kaplan–Meier analysis. (C) Diagram showed that LEF1 bound to the promoter of ID1 and putative LEF1 binding sites are indicated. (D) ChIP assays confirmed that LEF1 could bind to the ID1 promoter in ECA109 and TE1 cells. Quantification of immunoprecipitated DNA was shown by qRT-PCR. (E) Correlation of expression levels of LEF1 and ID1 in 98 patients (left panel) and total 129 patients (right panel) are shown. (PDF 238 kb) [file 13046_2019_1296_MOESM1_ESM.pdf]
