# Supplementary material for: The transcription factor LEF1 promotes tumorigenicity and activates the TGF-β signaling pathway in esophageal squamous cell carcinoma
Source: J Exp Clin Cancer Res. 2019 Jul 11;38:304. doi: 10.1186/s13046-019-1296-7 (PMC6625065; doi:10.1186/s13046-019-1296-7)
Supplement: Supplementary file 2 — Table S1. Correlation between LEF1 expression and clinicopathological characteristics in 243 patients. Table S2. Correlation between LEF1 expression and clinicopathological characteristics in total 338 patients. (DOCX 21 kb) [file 13046_2019_1296_MOESM2_ESM.docx]

**Additional file 1: Table S1** Correlation between LEF1 expression and clinicopathological

characteristics in 243 patients

|  | LEF1 | | |
| --- | --- | --- | --- |
|  | LEF1 high (159) LEF1 low (84) *P* value^a^ | | |
| Sex |  | | |
| Male | 135 | 75 | 0.432 |
| Female | 24 | 9 |  |
| Age(year)^b^ |  |  |  |
| > 65 | 37 | 17 | 0.63 |
| ≤65 | 122 | 67 |  |
| T_grade |  |  |  |
| 1 | 5 | 9 | **0.016** |
| 2 | 54 | 36 |  |
| 3 | 93 | 38 |  |
| 4 | 7 | 1 |  |
| Lymph node metastasis |  |  |  |
| N0 | 57 | 44 | 0.101 |
| N1 | 81 | 32 |  |
| N2 | 12 | 5 |  |
| N3 | 9 | 3 |  |
| TNM stage |  |  |  |
| I | 39 | 35 | **0.022** |
| II | 76 | 31 |  |
| III | 44 | 18 |  |
| Death |  |  |  |
| yes | 64 | 21 | **<0.01** |
| no | 95 | 63 |  |

a. Stactical signaficance was figrued out by chi-square test or Fisher's exact test

b. Data are presented as mean ± S.D.

**Additional file 1: Table S2** Correlation between LEF1 expression and clinicopathological

characteristics in total 338 patients

|  | LEF1 | | |
| --- | --- | --- | --- |
|  | LEF1 high (216) LEF1 low (122) *P* value^a^ | | |
| Sex |  | | |
| Male | 183 | 105 | 0.754 |
| Female | 33 | 17 |  |
| Age(year)^b^ |  |  |  |
| > 65 | 53 | 26 | 0.508 |
| ≤65 | 163 | 96 |  |
| T_grade |  |  |  |
| 1 | 16 | 26 | **<0.01** |
| 2 | 70 | 45 |  |
| 3 | 122 | 49 |  |
| 4 | 8 | 2 |  |
| Lymph node metastasis |  |  |  |
| N0 | 86 | 72 | **0.008** |
| N1 | 96 | 38 |  |
| N2 | 22 | 8 |  |
| N3 | 12 | 4 |  |
| TNM stage |  |  |  |
| I | 49 | 56 | **<0.01** |
| II | 98 | 40 |  |
| III | 69 | 26 |  |
| Death |  |  |  |
| yes | 89 | 26 | **<0.01** |
| no | 127 | 96 |  |

a. Stactical signaficance was figrued out by chi-square test or Fisher's exact test

b. Data are presented as mean ± S.D.
